# Arbeitsbedingungen in der Chirurgie und deren Auswirkungen: Ergebnisse einer nationalen Umfrage

**DOI:** 10.1007/s00104-024-02181-z

**Published:** 2024-10-10

**Authors:** J. Gumpp, F. Fritze-Büttner, B. Blank, S. Axt

**Affiliations:** 1 Themenreferat Familie und berufliche Perspektiven, Berufsverband der Deutschen Chirurgie e. V., Luisenstr. 58/59, 10117 Berlin, Deutschland; 2 Allgemeine Chirurgie/Schwerpunkt Allgemein- und Viszeralchirurgie, Kliniken im Naturpark Altmühltal, Klinik Eichstätt, Ostenstr. 31, 85072 Eichstätt, Deutschland; 3 Klinik für Allgemein- und Viszeralchirurgie, Sana Klinikum Lichtenberg, Fanningerstr. 32, 10365 Berlin, Deutschland; 4 Klinik für Plastische und Ästhetische Chirurgie/Handchirurgie, Dr. Erler Kliniken, Kontumazgarten 4–19, 90429 Nürnberg, Deutschland; 5 Klinik für Allgemeine, Viszeral- und Transplantationschirurgie, Universitätsklinikum Tübingen, Hoppe-Seyler-Str. 3, 72076 Tübingen, Deutschland

**Keywords:** Work-life Balance, Psyche, Familie, Zukunftsperspektive der Chirurgie, Status quo der Chirugie, Work-life balance, Mental health, Family, Future perspectives of surgery, Status quo of surgery

## Abstract

**Hintergrund:**

In chirurgischen Disziplinen werden schlechte Arbeitsbedingungen und eine hohe Unzufriedenheit der Chirurginnen und Chirurgen der verschiedenen Disziplinen aufgezeigt. Die psychischen Folgen dieser Bedingungen auf die Chirurginnen und Chirurgen selbst sowie Auswirkungen auf deren Familien sind bisher unzureichend beachtet worden.

**Ziel der Arbeit:**

Ziel dieser nationalen Umfrage des Berufsverbandes der Deutschen Chirurgie e. V. (BDC) war es, einen aktuellen Status der Arbeitsbedingungen in chirurgischen Abteilungen deutscher Kliniken zu erheben und die psychischen und familiären Auswirkungen dieser aufzuzeigen.

**Material und Methoden:**

Im Zeitraum 01–02/2024 wurde eine Umfrage mit 26 Fragen mit den Schwerpunkten psychische Belastung, beruflich bedingte partnerschaftliche und familiäre Probleme sowie Suchtverhalten an alle Mitglieder des BDC sowie an alle deutschen chirurgischen Fachgesellschaften geschickt.

**Ergebnisse:**

Es konnten 2221 Umfragen ausgewertet werden. Unter den Umfrageteilnehmern wurden Bürokratie (84,4 %) und ein unangemessener Ausgleich von Überstunden (68,1 %) als Hauptbelastung angesehen. Zur Bewältigung der Arbeitsbelastung wurden Alkohol (20,3 %), Nikotin (8,9 %) und Medikamente (8,3 %) angegeben; 60 % der Chirurginnen/Chirurgen gaben einen negativen Einfluss auf ihre Beziehung mit dem Partner/der Partnerin und 40 % auf die Beziehung mit den Kindern an.

**Diskussion:**

Von Chirurginnen/Chirurgen werden schlechte Arbeitsbedingungen angegeben. Diese haben massive Auswirkungen auf die Psyche der Chirurginnen/Chirurgen sowie auf deren familiäre Beziehungen. Konsekutiv denken viele Chirurginnen/Chirurgen daran, den chirurgischen Beruf aufzugeben. Um diese Umstände zu verbessern und den chirurgischen Beruf wieder attraktiver zu machen, muss ein drastisches Umdenken erfolgen.

**Zusatzmaterial online:**

Die Online-Version dieses Beitrags (10.1007/s00104-024-02181-z) enthält den in der Studie verwendeten Fragebogen.

## Abstract (en)

**Background:**

In surgical disciplines poor working conditions and a high level of dissatisfaction among surgeons in the various disciplines have been reported. The psychological consequences of these conditions on the surgeons themselves and the effects on their families have so far been inadequately considered.

**Objective:**

The objective of this national survey of the Professional Association of German Surgery (Berufsverband der Deutschen Chirurgie e. V.) was to determine the current status of working conditions in surgical departments of German hospitals and to demonstrate their psychological and familial effects.

**Material and methods:**

In the period January–February 2024 a questionnaire with 26 questions focusing on psychological stress, work-related partnership and family problems as well as addictive behavior was sent to all members of the Berufsverband der Deutschen Chirurgie e. V. and to all German surgical societies.

**Results:**

A total of 2221 questionnaires could be analyzed. Among the survey participants, bureaucracy (84.4%) and inadequate compensation for overtime (68.1%) were seen as the main stress factors. Alcohol (20.3%), nicotine (8.9%) and medication (8.3%) were cited as ways of managing the workload. Of the surgeons 60% reported a negative impact on their relationship with their partner and 40% on their relationship with their children.

**Discussion:**

The surgeons reported poor working conditions. These have a massive impact on the mental health of surgeons and on their family relationships. Consequently, many surgeons consider leaving the surgical profession. To improve this situation and to make the surgical profession attractive again, a drastic rethinking is needed.

## Hinführung zum Thema

In medizinischen Fachdisziplinen werden immer unattraktivere Arbeitsbedingungen sichtbar [14]. Neben einem zunehmend durch den demografischen Wandel bedingten wachsenden Versorgungsbedarf in Deutschland wird die aktuelle Ärztestatistik im Hinblick auf deren Entwicklung „sorgenvoll“ betrachtet [5]. Vor allem in chirurgischen Fachdisziplinen werden schlechte Arbeitsbedingungen und eine hohe Unzufriedenheit beschrieben [2, 19, 28]. Dies spiegelt sich auch in der Nachwuchsgewinnung wider. Eine Umfrage des „Berufsverbands der Deutschen Chirurgie e. V.“ (BDC) ergab, dass lediglich 18 % aller PJ-Studierenden erwägen, eine chirurgische Fachdisziplin zu wählen [24]. Ebenso zeigt eine Umfrage der Kassenärztlichen Bundesvereinigung (KBV), dass der Berufsstartwunsch in einer chirurgischen Fachdisziplin bei Studierenden von 35 % in der Vorklinik im Verlauf des Studiums drastisch auf 19,3 % sinkt; 36,5 % der Umfrageteilnehmer gaben sogar an, chirurgische Fachdisziplinen ganz ausgeschlossen zu haben [17]. Als Gründe dieses starken Rückganges des Berufswunsches Chirurgie werden durch Studierende maßgeblich die Vereinbarkeit von Beruf und Familie, welche 94,7 % der Studierenden als wichtigsten Faktor der Berufswahl angaben, sowie geregelte und flexible Arbeitszeiten, was für 84,1 und 83,8 % der Studierenden einen maßgeblichen Faktor darstellt, angegeben [17]. Als weitere Gründe für die geringe Nachfrage werden überlange Dienstzeiten und ein hohes Stresslevel während der täglichen Arbeit aufgeführt [27]. So nehmen zudem Faktoren wie „Aufstieg und Anerkennung“, die Studierende zu Beginn des Studiums für chirurgische Fachdisziplinen begeistern können, im Verlauf des Studiums signifikant zugunsten von „flexiblen Arbeitszeiten“ ab [30]. Durch Studierende werden in chirurgischen Disziplinen die höchste Prävalenz von Disstress und von über 80 % dieser ungünstige Arbeitsbedingungen angegeben [2]. Neben den enormen Schwierigkeiten der Nachwuchsgewinnung steht die stetig zunehmende Zahl der Ärztinnen und Ärzte über 60 Jahre, die in Deutschland aktuell etwa 23 % beträgt und auf eine zeitnahe weitere drastische Verschärfung der Arbeitssituation hinweist [5]. Demgegenüber steht die stete Zunahme stationärer chirurgischer Behandlungen. Diese lag im Jahr 2000 noch bei 4,1 Mio. chirurgischer Eingriffe von insgesamt 14,7 Mio. stationären Behandlungen und stieg bis zum Vor-Corona-Jahr 2019 auf 7,1 Mio. chirurgische Eingriffe bei insgesamt 18,8 Mio. stationären Behandlungen. Dabei sank die durchschnittliche Krankenhausverweildauer in chirurgischen Abteilungen von durchschnittlich 12,6 Tagen im Jahr 2000 auf 5,3 Tage, und damit auf weniger als die Hälfte, im Jahr 2022 [10].

## Einleitung

Die genannten Faktoren, bestehend aus einem sich aggravierenden Nachwuchsmangel, dem Ausscheiden der Babyboomer-Generation sowie einer stetig steigenden chirurgischen Fallzahl bei gleichzeitig kürzeren Krankenhausverweildauern führen zu einer immer weiter steigenden Arbeitsbelastung für Chirurginnen und Chirurgen mit direkten Auswirkungen auf die berufliche Zufriedenheit. Der BDC hat bereits im Jahr 2021 eine Erhebung der Zufriedenheit mit dem Fokus auf die Arbeitssituation durchgeführt, an der insgesamt 1940 Chirurginnen und Chirurgen teilgenommen haben [12]. Dies entsprach einem Anteil von etwa 8 % aller in Deutschland tätigen Chirurginnen und Chirurgen. In dieser Umfrage gaben 49,3 % an, bereits darüber nachgedacht zu haben, den chirurgischen Beruf aufgrund der Arbeitsbelastung aufzugeben. Vor allem durch Bürokratie und Administration sahen sich 39 % stark und 33,8 % häufig belastet. Mit deutlichem Abstand hiernach wurden Dienste und Überstunden als weitere Belastungsfaktoren angesehen, obwohl überwiegend 6 bis 10 (33,5 %) gefolgt von 1 bis 5 (27,4 %) Überstunden pro Woche und 4 bis 6 (33,8 %) gefolgt von 7 bis 9 (23,1 %) Dienste im Monat durch die Umfrageteilnehmenden angegeben wurden. Die Vereinbarkeit von Beruf und Privatleben wurde von insgesamt 62,7 % als unzufriedenstellend angesehen. Hierbei gaben 44,5 % der Chirurginnen und 39,2 % der Chirurgen diese als befriedigend und 22,3 % der Chirurginnen und 21,3 % der Chirurgen diese als schlecht bis sehr schlecht an. Um diese Arbeitsbelastung zu senken, gaben 32,2 % der Chirurginnen und 8,1 % der Chirurgen an, bereits in Teilzeit zu arbeiten. Die Hauptgründe hierfür waren bei Chirurgen mit 23,5 % eine zu hohe berufliche Belastung und mit 21,6 % eine durch die Arbeitszeitreduktion bedingte bessere Work-Life-Balance. Chirurginnen hingegen gaben mit 60,9 % die Kinderbetreuung als Grund der Teilzeit an. Um eine bessere Vereinbarkeit von Beruf und Familie zu erreichen, konnten sich bei dieser Umfrage jedoch 65,7 % der Chirurginnen und 51,6 % der Chirurgen vorstellen, eine gewisse Zeit in Teilzeit zu arbeiten. Als Hauptgrund der unzufriedenstellenden Vereinbarkeit von Beruf und Familie wurde die hohe Arbeitsbelastung angesehen. Um Angestellte vor einer Arbeitsüberlastung zu schützen, wurde das Arbeitszeitgesetz eingeführt. Bei der Umfrage gaben jedoch lediglich 16,6 % der Chirurginnen und Chirurgen an, dass das Arbeitszeitgesetz bei ihnen immer, und 36 %, dass es oft eingehalten werde; 20,7 % gaben an, dass es manchmal, 16,1 %, dass es selten, und 10,6 %, dass es nie eingehalten werde.

Auch frühere Studien zeigten bereits den Trend der Unzufriedenheit von Chirurginnen und Chirurgen mit den Arbeitsbedingungen auf [16, 21]. Dies stellt jedoch kein deutsches Phänomen dar, sondern besteht weltweit. Bei der Erhebung der beruflichen Charakteristika des chirurgischen Berufes zeigte sich, dass dieser von Chirurginnen und Chirurgen als höchst ethisch, äußerst relevant für Patientinnen und Patienten und mit direkter Rückmeldung, bedingt durch das chirurgische Outcome, angesehen wird. Jedoch weist die Chirurgie im Vergleich zu anderen medizinischen Berufsgruppen eine deutlich geringere Autonomie auf, was zu einer erhöhten Gefahr eines Burn-outs führen kann [18]. Burn-out, bedingt durch Unzufriedenheit mit der Arbeitssituation, stellt neben den Auswirkungen der Arbeit auf das Privatleben und der Dienstbelastung Prädiktoren für eine unzufriedenstellende Arbeitssituation dar. Eine hohe Arbeitszufriedenheit konnte demgegenüber durch geschützte Forschungszeit, gute Teambeziehungen, Begeisterung für die Arbeit und eine klinische Autonomie erzielt werden [25]. Eine amerikanische Umfrage hat ergeben, dass trotz Verbesserungen der beruflichen Rahmenbedingungen weiterhin geschlechtsspezifische Diskriminierungen und Nachteile in Bezug auf die Vereinbarkeit von Beruf und Familie bestehen. In dieser Studie gaben 21 % der Chirurginnen und 13 % der Chirurgen an, den Beruf nicht mehr zu wählen, wenn sie dies erneut müssten [26].

**Abb. 1:**
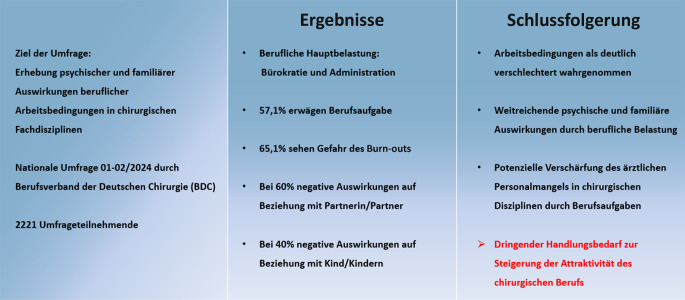
Arbeitsbedingungen in der Chirurgie und deren Auswirkungen. Ergebnisse einer nationalen Umfrage

Aktuelle Daten zu den tatsächlichen Arbeitsbedingungen von Chirurginnen und Chirurgen in Deutschland und deren psychische, aber auch familiäre Auswirkungen wurden in diesem Maße nicht erhoben und stehen daher nur unzureichend zur Verfügung. Um hier einen Status quo zu erheben und diese beruflichen Belastungen auf Chirurginnen und Chirurgen sowie auf deren Familien zu eruieren, wurde eine deutschlandweite Umfrage durchgeführt (Abb. 1).

## Material und Methoden

Um die Faktoren der beruflichen Zufriedenheit und der Lebensumstände von Chirurginnen und Chirurgen in Deutschland zu generieren, wurde durch den BDC 01–02/2024 eine nationale Umfrage durchgeführt, die auf die 2021 durchgeführte Umfrage aufbaut und diese um Fragen zur eigenen psychischen Belastung, deren Auswirkungen, beruflich bedingte partnerschaftliche und familiäre Probleme sowie Suchtverhalten ergänzt.

Die initiale nationale Umfrage 2021 umfasste hierbei 118 Fragen mit Mehrfachantwortmöglichkeiten, die von 1940 BDC-Mitgliedern oder Mitgliedern der deutschen chirurgischen Fachgesellschaften beantwortet wurden.

Der aktuelle Fragebogen umfasste 26 Fragen (s. Zusatzmaterial online) mit teilweise Einfach- und teilweise Mehrfachantworten.

## Ergebnisse

Der Fragebogen wurde an alle 15.075 BDC-Mitglieder verschickt. Insgesamt konnten 2221 Umfrageergebnisse eingeschlossen werden. Dies entspricht einer Umfrageteilnahme von 14,7 % aller BDC-Mitglieder und 5,4 % (Chirurgen: 3,4 %; Chirurginnen 8,6 %) aller in Deutschland tätigen Chirurginnen und Chirurgen [5].

**Abb. 2:**
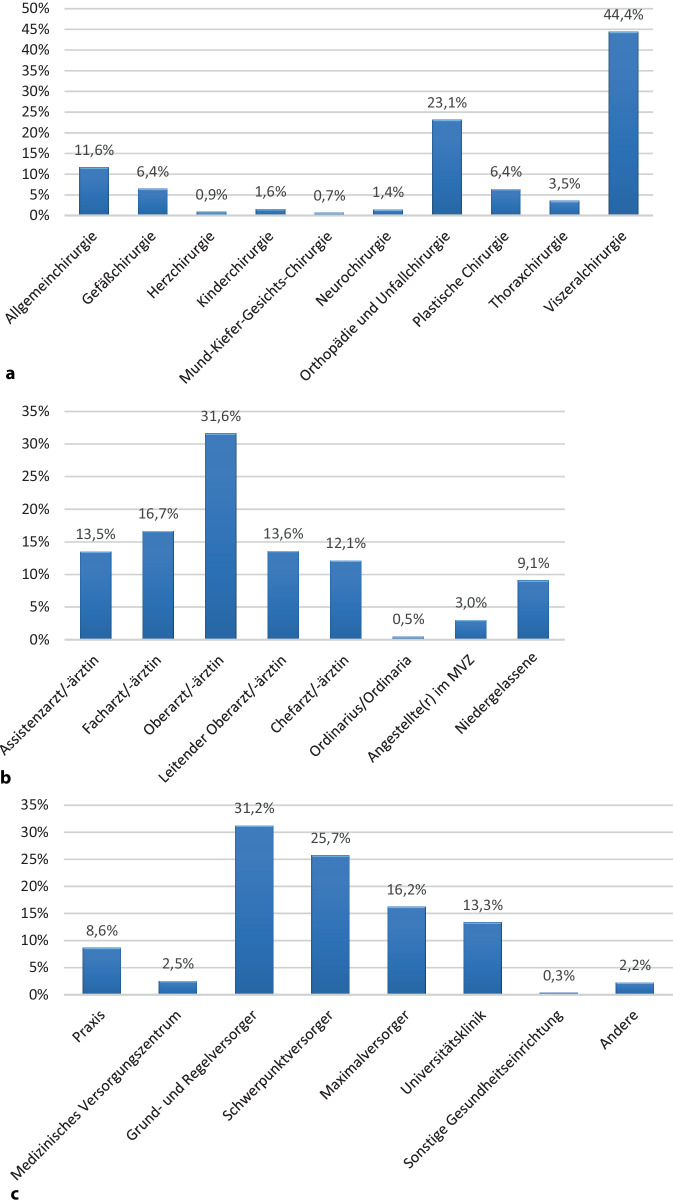
Demografische Daten der Umfrageteilnehmenden: **a** Fachdisziplin. **b** Funktion. **c** Versorgungstyp

Es waren 55,1 % der Umfrageteilnehmer männlich, 44,6 % weiblich und 0,3 % divers. Der Anteil der Chirurgen sank damit bei dieser Umfrage von 65,8 % bei der 2021 durchgeführten Umfrage; 2,9 % der Umfrageteilnehmer waren 20 bis 29 Jahre, 24,4 % 30 bis 39 Jahre, 27 % 40 bis 49 Jahre, 28,7 % 50 bis 59 Jahre und 16,9 % über 60 Jahre alt; 77,8 % gaben an, in einer Lebensgemeinschaft oder Ehe zu leben, wobei 60,2 % einen nichtmedizinischen und 37,6 % einen medizinischen Partner/Partnerin haben; 2,5 % waren geschieden und 7,8 % geschieden und erneut verheiratet; 9,4 % waren zum Zeitpunkt der Umfrage ledig; 28,1 % der Umfrageteilnehmenden hatten keine Kinder, während 17,3 % 1 Kind, 35,5 % 2 Kinder, 13,3 % 3 Kinder und 5,8 % 4 oder mehr Kinder angaben. Die demografischen Daten bezüglich Fachdisziplin, Funktion und Versorgungstyp des Arbeitsgebers sind in Abb. 2 angegeben.

Mit der beruflichen Tätigkeit insgesamt war die Mehrheit der Umfrageteilnehmenden zufrieden (40,5 % eher zufrieden, 23,8 % sehr zufrieden), während 11,8 % eher unzufrieden und 2,7 % gar nicht zufrieden waren. Bezüglich der Unzufriedenheit gab es keine wesentlichen Unterschiede bei Chirurginnen (14,6 %) und Chirurgen (14,4 %), während mehr Chirurgen (66,2 %) als Chirurginnen (61,8 %) mit der Arbeit zufrieden waren.

Bei der Betrachtung einzelner Faktoren fällt auf, dass Chirurginnen im Verlgeich zu Chirurgen bezüglich der beruflichen Chancengleichheit deutlich negativere Ansichten haben. Diesbezüglich zeigten sich 45,2 % der Chirurginnen unzufrieden, während dies lediglich 17 % der Chirurgen tun. Vielmehr zeigen sich 53,7 % der Chirurgen mit der Chancengleichheit zufrieden, wohingegen lediglich 12 % der Chirurginnen hier eine Zufriedenheit angeben.

**Abb. 3:**
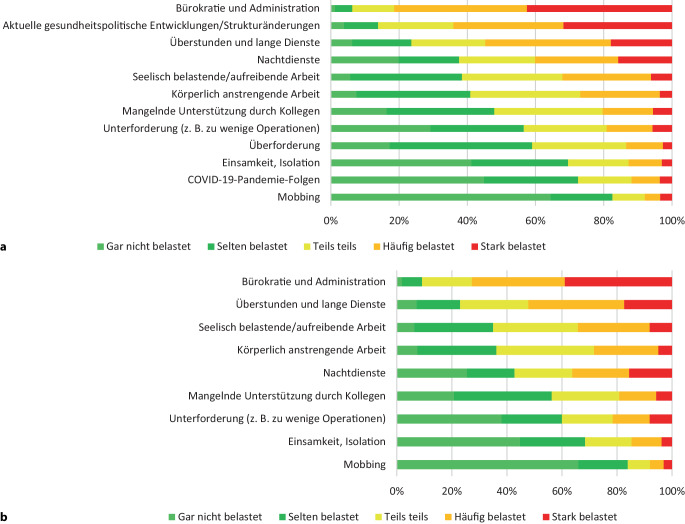
Angaben zur Arbeitsbelastung in den Umfragen 2021 und 2024. **a** Ergebnisse der Umfrage 2024. **b** Ergebnisse der Umfrage 2021. (modifiziert nach Fritze-Büttner F et al. [12])

Es würden 36,9 % der Umfrageteilnehmenden auch unter diesen Umständen wieder den chirurgischen Beruf wählen; 54,7 % gaben an, den Beruf nur unter anderen Rahmenbedingungen ausführen zu wollen. Diese bestünden vorwiegend in der Reduktion der Bürokratie (84,4 %), im angemessenen Ausgleich von Überstunden (68,1 %), der Reduktion von Diensten (57 %) und einer flexibleren Arbeitszeitgestaltung (56,5 %). Dies spiegelt die angegebene berufliche Hauptbelastung wider, die zu 81,4 % in Bürokratie und Administration gesehen wird [11]. Diese sehen Chirurgen (47,2 %) deutlich häufiger als Chirurginnen (37,1 %) als stark belastend an. Erst hiernach folgen mit 54,8 % Überstunden und lange Dienste, wobei dies Chirurginnen (59,9 %) im Vergleich zu Chirurgen (51,6 %) vermehrt als häufig oder stark belastend empfinden. Die Hauptbelastung in Bürokratie und Administration stieg damit bei dieser Umfrage in kurzer Zeit deutlich von 72,8 % in 2021 auf aktuell 81,4 %. Überstunden und lange Dienste wurden in beiden Umfragen etwa gleich häufig als belastend bewertet (52 % in 2021) (Abb. 3). Diesbezüglich gibt die Mehrheit der Umfrageteilnehmenden in der aktuellen Umfrage an, 6 bis 10 (34,9 %) gefolgt von 1 bis 5 (29,8 %) wöchentliche Überstunden sowie 4 bis 6 (31,3 %) gefolgt von 7 bis 9 (26,8 %) monatliche Dienste zu leisten [11].

Der Anteil der Chirurginnen und Chirurgen, der bereits erwogen hat, den Beruf aufzugeben, steigerte sich allerdings von 49,3 % 2021 auf nun 57,1 % drastisch [11]. Dies wurde dabei von deutlich mehr Chirurginnen (65,5 %) als von Chirurgen (50,2 %) erwogen. Nicht nur in Bezug auf das Geschlecht, sondern auch auf die berufliche Funktion bestehen diesbezüglich deutliche Unterschiede. So gaben 69 % der Ärztinnen/Ärzte in Weiterbildung an, einen Berufswechsel in Erwägung zu ziehen, sowie 63,8 % der Fach‑/Oberärztinnen und Fach‑/Oberärzte. Bei leitenden Oberärztinnen/Oberärzten wurde dies mit 51,2 % seltener und bei Chefärztinnen/Chefärzten mit 36,5 % am seltensten angegeben. Weiter gaben 87,3 % der Umfrageteilnehmenden an, Chirurginnen oder Chirurgen zu kennen, die bereits den Beruf aufgegeben bzw. gewechselt haben. Auch hier gaben dies mehr Chirurginnen (90,1 %) als Chirurgen (84,8 %) an.

Neben den bereits bestandenen Belastungen wird auch eine Verschärfung der Arbeitssituation bei 46,1 % der Umfrageteilnehmenden durch die SARS-CoV-2-Pandemie gesehen, während 43,1 % hier keine Verschärfung bemerkten. Hier gaben mehr Chirurgen (48,8 %) als Chirurginnen (42,5 %) eine Veränderung an. Ärztinnen und Ärzte in Weiterbildung empfanden am seltensten (37,1 %) eine Verschärfung, während leitende Oberärztinnen/Oberärzte (50 %) und Chefärztinnen/Chefärzte (59,6 %) dies deutlich häufiger empfanden. Am häufigsten wurde diese Verschärfung bei Maximalversorgern (52,6 %) angegeben, bei Universitätsklinika mit 34,1 % am wenigsten.

Es gaben 18,4 % der Umfrageteilnehmenden an, bereits aufgrund schwieriger oder unerträglicher beruflicher Situationen krankgeschrieben gewesen zu sein. Dabei erfolgte dies bei Chirurginnen häufiger (22,6 %) als bei Chirurgen (15,2 %). Ärztinnen/Ärzten in Weiterbildung gaben dies am häufigsten (25 %) an, während Chefärztinnen/Chefärzte (11,3 %) und niedergelassene Ärztinnen/Ärzte (10,5 %) dies deutlich seltener angaben. Insgesamt 15,2 % der Chirurginnen und Chirurgen gaben an, bereits aus diesen schwierigen oder unerträglichen Gründen eine Beratung oder Therapie beansprucht zu haben. Dies wurde 2021 mit noch 12,7 % angegeben und hat somit innerhalb kurzer Zeit einen deutlich gesteigerten Bedarf gefunden. Vor allem Chirurginnen (19,5 %) beanspruchen dies, während Chirurgen dies deutlich seltener (11,9 %) taten. Vergleichbar selten taten dies Chefärztinnen/Chefärzte (12,6 %) sowie niedergelassene Ärztinnen/Ärzte (12,8 %), während der Bedarf bei Ärztinnen/Ärzten in Weiterbildung (16,4 %) und Fach‑/Oberärztinnen und Fach‑/Oberärzten (16,3 %) höher lag; 65,1 % sahen bei sich selbst und 85 % bei Kolleginnen und Kollegen die Gefahr eines Burn-outs bestehen. Chirurginnen sehen die Gefahr eines Burn-outs bei sich (72,3 %) und bei Kolleginnen und Kollegen (90 %) deutlich höher als Chirurgen, die dies bei sich zu 59,9 % und bei Kolleginnen und Kollegen zu 81,4 % sehen. Ärztinnen/Ärzte in Weiterbildung (69,5 %), Fach‑/Oberärztinnen und Fach‑/Oberärzte (68 %) und leitende Oberärztinnen/Oberärzte (66,7 %) sehen diese Gefahr bei sich ähnlich hoch, während im Vergleich lediglich 52,2 % der Chefärztinnen/Chefärzte dies bei sich sahen.

Als weitere Folge dieser beruflichen Rahmenbedingungen wurde von 20,3 % der Umfrageteilnehmenden angegeben, dass Alkohol eine gewisse Rolle zur Bewältigung der Arbeitsbelastung und des psychischen Drucks spielt. Hier sind die Zahlen bei Chirurginnen (19,6 %) und Chirurgen (21,2 %) vergleichbar. Die höchste Quote ergab sich bei den leitenden Oberärztinnen/Oberärzten mit 23,6 %. Rauchen spielt im Vergleich zum Alkohol eine weniger relevante Rolle, da hier im Vergleich nur 8,9 % eine gewisse und 3,5 % eine große Rolle angaben, wobei auch hier Chirurginnen (10,8 %) eine niedrigere Relevanz im Vergleich zu Chirurgen (13,7 %) äußerten. Auch hier spielte das Rauchen am häufigsten bei leitenden Oberärztinnen/Oberärzten mit 4,3 % eine große und mit 11,6 % eine gewisse Rolle. Insgesamt gaben in Bezug auf die Rolle von Medikamenten 8,4 % an, dass dies eine gewisse Rolle zur Bewältigung des Arbeitsalltags spielt. Dabei zeigte sich bei Chirurginnen mit 10,2 % im Vergleich zu Chirurgen mit 7 % die häufigere Notwendigkeit. Die höchste Rate lag bei Ärztinnen/Ärzten in Weiterbildung, die hier eine gewisse Rolle mit 12,9 % angaben. In Bezug auf die Notwendigkeit der Einnahme dieser Produkte zur Bewältigung der Arbeitsbelastung wurden in Bezug auf Kolleginnen und Kollegen der Umfrageteilnehmenden höhere Werte angegeben (Alkohol: 34,8 %; Rauchen: 42,4 %; Medikamente 12,6 %). Vor allem Ärztinnen/Ärzte in Weiterbildung sahen bei Kolleginnen und Kollegen beim Rauchen (69,9 %) und bei Alkohol (50,8 %) eine Relevanz bei der Bewältigung der Arbeitsbelastung.

Besonders alarmierend ist, dass 26,8 % der Umfrageteilnehmenden angaben, Fälle von Suizid oder Suizidabsicht unter Kolleginnen und Kollegen zu kennen [13]. Chirurginnen gaben dies seltener (22,8 %) als Chirurgen (30,2 %) an. Vor allem leitende Oberärztinnen/Oberärzte (34,5 %) und Chefärztinnen/Chefärzte (40,9 %) äußerten diesbezüglich die Kenntnisnahme während Ärztinnen/Ärzte in Weiterbildung (16,4 %) damit am wenigsten Berührungspunkte hatten. In Bezug auf die Versorgungstypen wurde dies v. a. bei Maximalversorgern (30,2 %) und bei niedergelassenen Ärztinnen/Ärzten (30,9 %) angegeben.

**Abb. 4:**
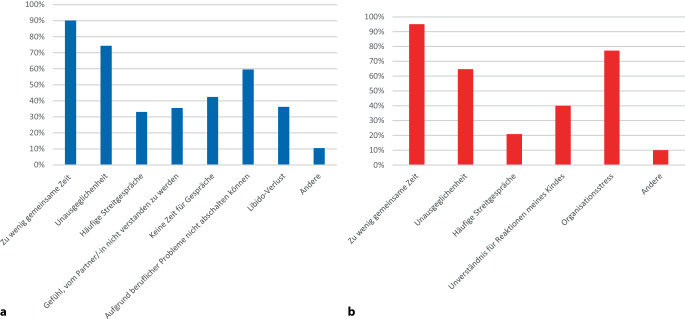
Einfluss der beruflichen Belastungen auf die Beziehung mit dem Partner/der Partnerin sowie mit den Kindern. **a** Einfluss der beruflichen Belastungen auf die Beziehung mit dem Partner/der Partnerin. **b** Einfluss der beruflichen Belastungen auf die Beziehung mit den Kindern

Die Umfrageteilnehmenden sahen auch eine erhebliche beruflich bedingte Belastung in Bezug auf das Privatleben und die familiäre Situation. Knapp 60 % äußerten eine negative Auswirkung auf ihre Beziehung mit dem Partner/der Partnerin. Chirurginnen (62,9 %) und Chirurgen (57,9 %) äußerten dies etwa gleichermaßen. Jedoch ergab die Umfrage, dass v. a. Ärztinnen/Ärzte in Weiterbildung mit 75,4 % deutlich häufiger diese Auswirkung auf das Privatleben und die familiäre Situation sahen. In den Kliniken wird diese Belastung v. a. in den Universitätsklinika (65,9 %) empfunden, während niedergelassene Ärztinnen/Ärzte diese am seltensten (47,7 %) angaben. Als wesentliche Faktoren, die die erhebliche Belastung bedingen, wurden zu wenig gemeinsame Zeit (90,1 %), Unausgeglichenheit (74,3 %) und die Unfähigkeit, abschalten zu können, angegeben (Abb. 4).

So gaben letztlich 22,6 % der Umfrageteilnehmenden an, aufgrund der beruflichen Belastungssituation eine partnerschaftliche Trennung durchgemacht zu haben. Die betraf Chirurginnen (21,2 %) und Chirurgen (23,8 %) etwa gleichermaßen. Am häufigsten gaben leitende Oberärztinnen/Oberärzte (26,4 %) und Ärztinnen und Ärzte in Universitätsklinika (25,4 %) Trennungen aufgrund der beruflichen Belastung an. Am wenigsten davon betroffen waren Ärztinnen/Ärzte in Weiterbildung (19,9 %) und niedergelassene Ärztinnen/Ärzte (20,9 %). Zudem gaben 10,3 % der Umfrageteilnehmenden an, geschieden zu sein, wobei mehr Chirurgen (12,4 %) als Chirurginnen (7,6 %) hiervon betroffen waren. Am häufigsten wurde eine Scheidung von leitenden Oberärztinnen/Oberärzten (15,5 %), von Chefärztinnen/Chefärzten (15,7 %) sowie von niedergelassenen Ärztinnen/Ärzten (15,7 %) angegeben.

Rund 79 % der Umfrageteilnehmenden waren Eltern. Hiervon sahen etwa 40 % eine erhebliche berufsbedingte Belastung in Bezug auf die Beziehung mit dem Kind/den Kindern. Dies wurde von Chirurgen (45,7 %) wesentlich häufiger angegeben als von Chirurginnen (32,8 %). Ärztinnen/Ärzte in Weiterbildung sahen dies mit 24,6 % am seltensten vorliegend, wohingegen leitende Oberärztinnen/Oberärzte (46,9 %) und niedergelassenen Ärztinnen/Ärzte (4,4 %) dies als wesentlich häufigeres Problem angaben. Eltern, die in Universitätsklinika angestellt sind, sahen diese negativen Auswirkungen mit 34,9 % im Vergleich ebenfalls seltener.

## Schlussfolgerung

Bereits in der Umfrage 2021 konnte eine erhebliche berufliche Belastung von Chirurginnen und Chirurgen aufgezeigt werden. Hier standen bereits die überbordende Bürokratie und Administration im Vordergrund. Diese wird im kurzfristigen Verlauf als deutlich belastender wahrgenommen, während physische Belastungen wie Überstunden und Dienste in beiden Umfragen als etwa gleich belastend beurteilt wurden. Somit stellt sich weiterhin die Frage, wie Bürokratie und administrative Aufgaben effizient abgebaut werden können. Lösungsansätze bestehen in Stationsassistierenden, die durch die Übernahme nichtmedizinischer Aufgaben eine entlastende Rolle darstellen können. Weitere Entlastung kann z. B. durch Kodierkräfte und Fallmanager erreicht werden, die organisatorische Aufgaben unter Supervision des ärztlichen Personals durchführen. Jedoch kann die Einführung von immer mehr Berufsgruppen wie den Stationsassistierenden, Servicekräften, Ernährungs- und Wundmanagern durch immer mehr Schnittstellen und einen immer höheren Koordinationsaufwand kontraproduktiv sein und zu einer Steigerung der administrativen Aufgaben führen [6]. Zudem kann durch die Vielzahl der Behandelnden und die damit einhergehende Verlagerung der Behandlung in Einzelaspekte eine Behandlungsdiffusion stattfinden, bei der sich letztendlich niemand mehr für die Patientin/den Patienten im Gesamten verantwortlich fühlt [23]. Demgegenüber steht, dass eine gut organisierte Station mit funktionierenden Abläufen zu einer relevanten Entlastung der Mitarbeiter und damit zu einer Senkung der Belastung und Steigerung der Mitarbeiterzufriedenheit führt [7]. Eine Personalsteigerung nichtärztlich Mitarbeitender ist sicherlich sinnvoll, jedoch müssen die genannten kritischen Punkte bei einem Ausbau dieser Möglichkeit berücksichtigt werden.

Die beruflich bedingte Belastung wirkt sich sowohl auf das Privatleben von Chirurginnen und Chirurgen selbst als auch auf die Beziehung zu Partnern und Kindern aus. Starre und unflexible Arbeitszeitmodelle spielen hier in Bezug auf die Vereinbarkeit von Beruf und Familie eine wesentliche Rolle. Aber auch nicht oder schlecht planbare Überstunden beeinflussen diese Vereinbarkeit sehr stark. In der aktuellen sowie in der 2021 durchgeführten Umfrage wurden von der Mehrheit der Chirurginnen und Chirurgen 6 bis 10 Überstunden pro Woche und 4 bis 6 Dienste im Monat angegeben. Sowohl bei den Überstunden als auch bei den Diensten zeigte sich eine leichte Zunahme der Belastung. Gerade Überstunden stellen eine nicht planbare kurzfristige Veränderung der Arbeitszeit dar, die zu einem hohen Frustrationspotenzial führen kann. Sicherlich auch hierdurch bedingt, konnte sich bereits 2021 die Mehrheit der Umfrageteilnehmenden vorstellen, eine Arbeitszeitreduktion durch die Inanspruchnahme von Teilzeit zu erreichen. Im Jahr 2010 lag der Anteil an Chirurginnen und Chirurgen, die in Teilzeit arbeiteten, noch bei etwa 10 % [9, 12]. Dieser Trend ist deutlich zunehmend und muss von Arbeitgebern immer häufiger berücksichtigt werden. Die Inanspruchnahme einer Teilzeitbeschäftigung wurde auch in einer anderen Umfrage aufgezeigt, bei der auch der in Zukunft weiter steigende Anteil aufgezeigt wurde [29]. Ein positiver Einfluss der Arbeitsreduktion in anderen medizinischen Berufsgruppen konnte bereits nachgewiesen werden. So zeigten Internistinnen und Internisten in Teilzeitbeschäftigung ein besseres Wohlbefinden, eine bessere psychische Gesundheit sowie weniger depressive Symptome [4]. Eine weitere Langzeitstudie konnte eine Verbesserung der Vereinbarkeit von Beruf und Privatleben aufzeigen. Eine Reduktion des Burn-outs konnte hier jedoch nicht nachgewiesen werden [3]. Jedoch können diesbezüglich ggf. andere Arbeitsmodelle, wie z. B. ein Gleitzeitmodell mit flexibleren Arbeitszeiten oder dem Angebot verschiedener Dienstmodelle mit Kurzdiensten oder langen Nachtdiensten, die Vereinbarkeit von Beruf und Privatleben bzw. Familie verbessern [31]. Auch im Moment noch unkonventionell erscheinende Konzepte wie die 4‑Tage-Woche, eine Erhöhung des Urlaubsanspruchs oder variable Arbeitszeitmodelle könnten diesen Effekt bewirken [1, 15].

Die aktuellen Arbeitsbedingungen in chirurgischen Disziplinen führen jedoch dazu, dass Chirurginnen und Chirurgen mehrheitlich (knapp 60 %) mit dem Gedanken spielen, den Beruf zu wechseln bzw. aufzugeben, und das trotz einer großen Begeisterung für die chirurgische Profession; 87 % der Umfrageteilnehmenden kennen Kolleginnen und Kollegen, die den Beruf bereits aufgegeben haben und somit der Umfrage nicht mehr zugänglich waren. In einer Umfrage alle medizinischen Disziplinen betreffend liegt die Quote der Erwägung eines Berufswechsels bei lediglich 25 % und somit deutlich unter der in dieser Umfrage angegebenen [14]. Noch gravierender ist die Tatsache, dass weit über die Hälfte der Chirurginnen und Chirurgen bei sich selbst oder bei Kolleginnen und Kollegen die Gefahr eines Burn-outs sieht. Als relevante Risikofaktoren für die Entstehung eines Burn-outs werden in einer Übersichtsarbeit die Arbeitsanforderungen im medizinischen Beruf v. a. bei Berufsanfängern, Sorgen um die Patientenversorgung, eine geringe berufliche Autonomie, ein schlechtes Arbeitsumfeld und eine schlechte Work-Life-Balance aufgeführt [32]. Eine weitere Übersichtsarbeit mit rund 22.500 Ärztinnen und Ärzten konnte aufzeigen, dass die Rate von Ärztinnen und Ärzten mit Depressionen oder depressiven Symptomen bei etwa 30 % lag. Hier konnte zudem eine jährliche Steigerung dieser Rate von 0,5 % nachgewiesen werden [22]. Es können jedoch wirkungsvolle Präventionsmaßnahmen durchgeführt werden, die maßgeblich auf der Erkennung und Beseitigung von Stressursachen beruhen. Aber auch die Etablierung achtsamkeitsbasierter Praktiken, die Einführung von Stressbewältigungsmaßnahmen, wie z. B. Meditation, Yoga, Akkupunktur sowie die Förderung der positiven Einstellung, sowie die Etablierung organisatorischer Interventionen wie die Verringerung der Arbeitsbelastung und die Mitbestimmung der Arbeitsgestaltung fördern das Wohlbefinden, das Engagement und die Belastbarkeit von Mitarbeitenden und reduzieren das Burn-out-Risiko [8]. Mit derartigen Maßnahmen kann Burn-out reduziert werden und einem weiteren Voranschreiten der Auswirkungen dieser Belastungssituationen entgegengewirkt werden. Demgegenüber geben jedoch mehr als 25 % der Umfrageteilnehmenden an, Kolleginnen und Kollegen mit Suizidabsichten zu kennen oder Suizidfälle miterlebt zu haben. Bereits 2001 konnte aufgezeigt werden, dass die Suizidrate unter v. a. Ärztinnen, aber auch Ärzten im Vergleich zur Normalbevölkerung überdurchschnittlich hoch erscheint. Hier konnte auch eine höhere Prävalenz von psychiatrischen Erkrankungen und Suchtmittelabhängigkeiten bei Ärztinnen und Ärzten nachgewiesen werden [20]. Ursächlich hierfür wurden hier bereits die Arbeitsbedingungen benannt. So wurden das subjektive Gefühl des Kontrollverlustes im Berufsalltag und alltägliche Berufsverpflichtungen, die mit hohem emotionalem Stress verbunden sind, als wesentliche Faktoren benannt. Als Risikogruppen konnten Medizinerinnen und Mediziner in interdisziplinären Notfallaufnahmen, Chirurginnen und Chirurgen sowie Anästhesistinnen und Anästhesisten bestimmt werden. Diese in höchstem Maße alarmierende Entwicklung wurde in der Umfrage 2021 und in der aktuellen aufgezeigt und verdeutlicht, dass sich diese weiter aggraviert hat. Hier wurden bereits Forderungen zur Verbesserung der Arbeitsbedingungen formuliert. Trotz bekannter und benannter Problematik laufen Bemühungen der Berufsverbände, auf diese Bedingungen positiv einzuwirken, offensichtlich ins Leere. Die hier dargelegten Probleme haben nicht nur für die Chirurgie Gültigkeit [14], zeigen sich aufgrund der aktuellen politischen Entwicklungen und Unsicherheiten hier jedoch möglicherweise schneller.

Limitation dieser Umfrage könnte die Motivation der Umfrageteilnehmer sein. So ist nicht auszuschließen, dass unzufriedene Chirurginnen und Chirurgen eher an Umfragen zur Erhebung der Arbeitssituationen teilnehmen als zufriedene. Hier ist jedoch die hohe Fallzahl mit einem Anteil von etwa 15 % aller BDC-Mitglieder bzw. 5,4 % aller in Deutschland tätigen Chirurginnen und Chirurgen zu berücksichtigen. In Bezug auf die Altersverteilung sind die unter 30-Jährigen unterrepräsentiert. Die übrigen Altersgruppen sind ähnlich verteilt.

## Fazit für die Praxis

- Die aktuelle Umfrage zeigt erneut die Ursachen der Unzufriedenheit von Chirurginnen und Chirurgen mit der beruflichen Situation und deren Auswirkungen auf das Privatleben auf. Entgegen den Erwartungen einer Verbesserung konnte eine weitere Verschlechterung der Situation festgestellt werden. Diese wird alters- und geschlechtsunabhängig wahrgenommen und kritisiert. Unter diesen Bedingungen ist mit einer weiteren Verschärfung der Personalsituation durch vermehrte Berufswechsel zu rechnen. Zudem kann unter diesen Bedingungen nicht von einer suffizienten Nachwuchsgewinnung ausgegangen werden. Diese ist aber in Anbetracht der ausscheidenden Babyboomer-Generation und der steten Fallzahlsteigerung zur Aufrechterhaltung einer adäquaten Versorgungssicherheit in Deutschland dringend notwendig.
- Zur Verbesserung der Arbeitsbedingungen müssen Bürokratie und administrative Aufgaben deutlich reduziert werden, um Chirurginnen und Chirurgen zeitlich zu entlasten und vermehrt ihrer patientennahen Arbeit zuzuführen. Zudem müssen zukunftsfähige flexiblere Arbeitszeit- und Dienstzeitmodelle etabliert werden, die eine bessere Vereinbarkeit von Beruf und Familie gewähren. Ebenfalls könnte eine engere Anbindung der Familie an den Arbeitgeber durch z. B. Schaffung ausreichender Betreuungsplätze für Klein‑, Kindergarten- und Schulkinder die Vereinbarkeit von Beruf und Familie verbessern.
- Wenn es gelingt, den chirurgischen Beruf wieder attraktiver und zeitgemäßer zu machen, kann dies zu einer wesentlichen Steigerung der Zufriedenheit der Chirurginnen und Chirurgen und zu einer effizienteren Nachwuchsgewinnung führen. Sollten keine Verbesserungen erzielt werden können, könnte eine drastische Verschlechterung des Personalmangels durch zahlreiche Berufswechsel bzw. -aufgaben die Folge sein.

## Supplementary Information

In der Studie verwendete Fragebogen

## Data Availability

Die erhobenen Datensätze können auf begründete Anfrage in anonymisierter Form beim korrespondierenden Autor angefordert werden. Die Daten befinden sich auf einem Datenspeicher des Berufsverbandes der Deutschen Chirurgie e. V. (BDC).
