# Supplementary material for: Working conditions in surgery and their impact: Results of a national survey
Source: Chirurgie (Heidelb). 2024 Oct 10;96(8):657–66. [Article in German] doi: 10.1007/s00104-024-02181-z (PMC12307500; doi:10.1007/s00104-024-02181-z)
Supplement: Supplementary file 1 — In der Studie verwendete Fragebogen [file 104_2024_2181_MOESM1_ESM.pdf]

Abbildung 1: Fragebogen

1. Geben Sie Ihr Geschlecht an
    a. weiblich
    b. männlich
    c. divers

2. Geben Sie Ihr Alter an
    a. 20-29
    b. 30-39
    c. 40-49
    d. 50-59
    e. > 60

3. Geben Sie Ihren Familienstand an
    a. ledig
    b. in Lebensgemeinschaft lebend/ verheiratet
    c. geschieden und nicht in erneuter Partnerschaft lebend
    d. geschieden und erneut in Lebensgemeinschaft lebend/ verheiratet
    e. anderer Familienstand, bitte nennen: (Freitext………..)

4. Falls Sie einen Ehepartner/Lebenspartner heben: Beruf des derzeitigen Partners (bzw. letzter aktiver Beruf)
    a. Mediziner
    b. Nicht- Mediziner

5. Wieviel Kinder haben Sie: eigene, inkl. einer eventuellen Schwangerschaft, und Sorgeberechtigte/ Patchwork (Kinder leben im Haushalt)?
    a. 1
    b. 2
    c. 3
    d. 4
    e. > 4

6. Was ist Ihr derzeitiger Tätigkeitsbereich?
    a. Chirurgie aktiv
    b. Chirurgie, aber derzeit im Krankenstand, Mutterschutz oder Elternzeit
    c. Anderer medizinischer Beruf (nicht chirurgisch)
    d. Andere Tätigkeit (nicht medizinisch)
    e. Nicht berufstätig (Haushalt, arbeitslos, etc.)
    f. Im Ruhestand

7. In welchem chirurgischen Fachgebiet sind/ waren Sie hauptsächlich tätig, bzw. welche Facharztausbildung wollen Sie beginnen, sofern sie sich im Studium/ Praktischen Jahr befinden?
    a. Allgemeinchirurgie
    b. Gefäßchirurgie
    c. Herzchirurgie
    d. Kinderchirurgie
    e. Mund- Kiefer- Gesichtschirurgie
    f. Neurochirurgie
    g. Orthopädie und Unfallchirurgie
    h. Plastische Chirurgie
    i. Thoraxchirurgie
    j. Viszeralchirurgie

8. In welchem Krankenhaustyp sind/ waren Sie hauptsächlich tätig?
    a. Praxis
    b. Medizinisches Versorgungszentrum
    c. Krankenhaus der Grund- und Regelversorgung
    d. Krankenhaus der Schwerpunktversorgung
    e. Krankenhaus der Maximalversorgung
    f. Universitätsklinik
    g. Sonstige Gesundheitseinrichtung (Gesundheitsamt, MDK, etc.)
    h. Andere, bitte nennen: (Freitext………)

9. In welcher Funktionsstellung befinden Sie sich?
    a. Studentin/ Student
    b. Assistenzärztin/ Assistenzarzt
    c. Fachärztin/ Facharzt
    d. Oberärztin/ Oberarzt
    e. Leitende Oberärztin/ Leitender Oberarzt
    f. Chefärztin/ Chefarzt
    g. Ordinaria/ Ordinarius
    h. Angestellte/ Angestellter im MVZ
    i. Niedergelassene/ Niedergelassener

10. Wie zufrieden sind Sie mit folgenden Bereichen Ihrer beruflichen Tätigkeit?

	sehr zufrieden	eher zufrieden	teils-teils	eher unzufrieden	gar nicht zufrieden	Keine Angaben
mit Ihrer beruflichen Tätigkeit insgesamt						
mit der Arbeitsmenge						
mit der Arbeitszeitregelung						
mit der Weiterbildung						
mit Aufstiegs- und Entwicklungsmöglichkeiten (Förderung)						
mit Fortbildungsmöglichkeiten						
bezüglich Chancengleichheit im Beruf						

11. Wie sehr fühlen Sie sich in Ihrer beruflichen Situation belastet durch… ?

	Stark belastet	Häufig belastet	Teils- teils	Selten belastet	Gar nicht belastet
Seelisch belastende und aufreibende Arbeit					

Körperlich anstrengende Arbeit					
Überstunden und lange Dienste					
Nachtdienste					
Überforderung					
Unterforderung (z. B. zu wenige Operationen)					
Einsamkeit, Isolation					
Abhängigkeitsverhältnisse					
Chaotische Arbeitssituation					
Bürokratie und Administration					
Mangelnde Unterstützung durch Kollegen					
Mangelnde Unterstützung durch Vorgesetzte					
Mobbing					
Covid- 19- Pandemie-Folgen					
Aktuelle gesundheitspolitische Entwicklungen/ Strukturänderungen					

12. Wie viele **Überstunden** machen Sie **wöchentlich im Durchschnitt**?
    a. 0
    b. 1-5
    c. 6-10
    d. 11-15
    e. 15-20
    f. > 20

13. Wie viele Dienste leisten Sie monatlich (Anwesenheits- und Rufdienste)?
    a. 1-3
    b. 4-6
    c. 7-9
    d. 10-12
    e. >12

14. Dachten Sie schon jemals an die Gefahr eines Burnouts?

	Ja	Nein
…bei Ihnen selbst		
…bei Kolleg:innen		

15. Waren Sie schon jemals aufgrund einer schwierigen oder unerträglichen beruflichen Situation krankgeschrieben?
    a) Ja
    b) Nein

16. Haben Sie schon einmal aus beruflichen Gründen eine Therapie/ Beratung beansprucht?
    a) Ja
        a. Wenn ja, welche: (Freitext ……)
    b) Nein

17. Spielen die folgenden Mittel bei der Bewältigung der chirurgischen Arbeitsbelastung und des psychischen Drucks bei Ihnen eine Rolle?

	Große Rolle	Gewisse Rolle	Keine Rolle	Weiß nicht
Rauchen				
Alkohol				
Medikamente				
Drogen				

18. Spielen die folgenden Mittel bei der Bewältigung der chirurgischen Arbeitsbelastung und des psychischen Drucks bei Kolleg: innen eine Rolle?

	Große Rolle	Gewisse Rolle	Keine Rolle	Weiß nicht
Rauchen				
Alkohol				
Medikamente				
Drogen				

19. Haben Sie schon jemals daran gedacht, den Beruf der Chirurgin/ des Chirurgen aufzugeben?
    a) Ja
    b) Nein

20. Kennen Sie Chirurg: innen, die den Beruf gewechselt oder aufgegeben haben?
    a) Ja
        a. Wenn ja, Anzahl: (Freitext ……)

    b) Nein

21. Kennen Sie einen Suizidfall oder eine Suizidabsicht unter Chirurg: innen?
    a) Ja
    b) Nein

22. Würden Sie wieder Chirurgin/ Chirurg werden wollen?
    a. Ja auf jeden Fall
    b. Ja, aber unter anderen Bedingungen (Mehrfachnennungen möglich)
        i. Adäquate Arbeitszeiterfassung
        ii. Reduktion von Überstunden

iii. Angemessener Ausgleich von Überstunden (Freizeitausgleich/ Bezahlung)
iv. Reduktion von Diensten
v. Kein Schichtdienst
vi. Reduktion von Bürokratie und Administration
vii. Flexible Arbeitszeitgestaltung (angepasst an klinischen Alltag)
viii. Möglichkeiten der Teilzeittätigkeit

c. Nein, sicher nicht

23. Hat die Covid-19- Pandemie aus Ihrer Sicht Ihre Belastungssituation am Arbeitsplatz verschärft?
    a. Ja
    b. Nein
    c. Weiß nicht

24. Wirkt sich Ihre berufliche Belastungssituation negativ auf Ihre Beziehung aus?
    a. Ja
    Wenn ja: wie äußern sich diese (Mehrfachnennungen mgl.):
        i. Zu wenig gemeinsame Zeit (Freizeitgestaltung)
        ii. Unausgeglichenheit
        iii. Häufige Streitgespräche
        iv. Das Gefühl, von der Partnerin/ vom Partner nicht verstanden zu werden
        v. Keine Zeit für Gespräche
        vi. Nicht abschalten können, berufliche Probleme lassen mich zu Hause nicht zur Ruhe kommen
        vii. Libido- Verlust
        viii. Sonstiges (Freitext…)

    b. Nein
    c. Weiß nicht

25. Gab es während Ihrer beruflichen Laufbahn partnerschaftliche Trennungen, die Sie auf Ihre berufliche Belastungssituation zurückführen?
    a. Ja
    b. Nein
    c. Weiß nicht

26. Falls Sie ein Kind/ Kinder haben, wirkt sich Ihre berufliche Belastungssituation negativ auf die Beziehung zu ihrem Kind/ ihren Kindern aus?
    a. Ja
    Wenn ja: wie äußern sich diese (Mehrfachnennungen mgl.):
        i. Zu wenig gemeinsame Zeit
        ii. Unausgeglichenheit
        iii. Häufige Streitgespräche
        iv. Unverständnis für Reaktionen meines Kindes
        v. Organisationsstress
        vi. Sonstiges (Freitext…)
    b. Nein
    c. Weiß nicht
